# Inhibition of synovitis and joint destruction by a new single domain antibody specific for cyclophilin A in two different mouse models of rheumatoid arthritis

**DOI:** 10.1186/ar4401

**Published:** 2013-12-05

**Authors:** Li Wang, Junfeng Jia, Conghua Wang, Xiaokui Ma, Chenggong Liao, Zhiguang Fu, Bin Wang, Xiangmin Yang, Ping Zhu, Yu Li, Zhinan Chen

**Affiliations:** 1 Cell Engineering Research Centre, Cancer Biology of State Key Laboratory, Fourth Military Medical University, No. 17 West Changle Road, Xi’an 710032, Shaanxi, PR China; 2 Department of Clinical Immunology, First Affiliated Hospital, Fourth Military Medical University, No. 15 West Changle Road, Xi’an 710032, Shaanxi, PR China; 3 Department of Life Science, Shaanxi Normal University, Xi’an 710062, Shaanxi, PR China

## Abstract

**Introduction:**

Cyclophilin A (CypA) is implicated in rheumatoid arthritis (RA) pathogenesis. We studied whether a novel anti-CypA single domain antibody (sdAb) treatment would modulate the severity of the disease in two different animal models of RA.

**Methods:**

A novel sdAb, named sdAbA1, was screened from an immunized camel sdAb library and found to have a high binding affinity (K_D_ = 6.9 × 10^-9^ M) for CypA. The SCID-HuRAg model and the collagen-induced arthritis (CIA) in mice were used to evaluate the effects of sdAbA1 treatment on inflammation and joint destruction. For *in vitro* analysis, monocytes/macrophages were purified from synovial fluid and peripheral blood of patients with RA and were tested for the effect of anti-CypA sdAb on metalloproteinase (MMP) production. Human monocyte cell line THP-1 cells were selected and western blot analyses were performed to examine the potential signaling pathways.

**Results:**

In the CIA model of RA, the sdAbA1 treatment resulted in a significant decrease in clinical symptoms as well as of joint damage (*P* <0.05). In the SCID-HuRAg model, treatment with anti-CypA antibody sdAbA1 significantly reduced cartilage erosion, inflammatory cell numbers and MMP-9 production in the implanted tissues (*P* <0.05). It also significantly reduced the levels of human inflammatory cytokines IL-6 and IL-8 in mouse serum (*P* <0.05). No toxic effects were observed in the two animal models. *In vitro* results showed that sdAbA1 could counteract CypA-dependent MMP-9 secretion and IL-8 production by interfering with the ERK-NF-κB pathway.

**Conclusions:**

Blockade of CypA significantly inhibited synovitis and cartilage/bone erosion in the two tested animal models of RA. Our findings provide evidence that sdAbA1 may be a potential therapeutic agent for RA.

## Introduction

Rheumatoid arthritis (RA) is a chronic and debilitating disease of the joints characterized by synovial inflammation and progressive destruction of articular cartilage and bone [1]. The number of inflammatory cells and the level of inflammatory cytokines in the joints correlate with the extent of synovitis, and matrix metalloproteinases (MMPs) at the cartilage–pannus junction of RA patients are the main proteases involved in the invasion and degradation of cartilage [2]. In RA, the number of monocytes/macrophages, which secrete multiple cytokines [3] and MMPs, is significantly increased in both the lining and sublining areas of the RA synovium, where they play a critical role in inflammation and joint destruction.

Cyclophilins are a novel family of proteins exerting potent chemotactic capacity that have been well researched recently. Cyclophilins are widely expressed intracellular proteins, well known as receptors for the immunosuppressive drug cyclosporine A (CsA). Cyclophilin A (CypA) is the most abundant cyclophilin and can be actively released into extracellular tissue spaces in response to inflammatory stimuli [4]. Extracellular CypA is not only a strong *in vitro* chemoattractant for neutrophils, T cells and monocytes, but can also induce a rapid influx of leukocytes *in vivo*[5]. High levels of CypA have been detected in the serum and synovial fluid of RA patients and the amount of CypA was closely related to disease severity [6]. In RA synovium, CypA has been detected in most of macrophages in the lining layer and sublining area, and CypA staining overlaps with MMP-9 staining [7]. Our previous study claims that CypA upregulates MMP-9 expression and adhesion of monocytes/macrophages [8], and may aggravate cartilage erosion when injected *in vivo*[9]. Taken together, these results show that CypA plays an important role in the pathogenesis of RA and that reagents targeting CypA could be beneficial in the treatment of this disease.

Monoclonal antibodies have played vital roles in antibody-based therapies against various diseases including RA [10]. Nevertheless, difficulties such as the costs of production and protein stability can be encountered. Recent major progress involved the use of single-domain antibodies (sdAbs) derived from camelids. In addition to conventional antibodies, camelids produce antibodies composed of heavy chains only, with a single variable domain (referred to as VHH) capable of recognizing specific antigens. These variable domains, named sdAbs, are the smallest naturally occurring, intact antigen-binding units and are highly valuable for their unique features. SdAbs are easily produced by bacterial fermentation. This approach implies an estimated 10-fold reduction in production costs as compared with conventional therapeutic antibodies, which are all produced in mammalian systems [11]. The long CDR3 in sdAbs recognizes structures such as pockets and clefts on the surface of antigens that are inaccessible for conventional antibodies. Other attractive features of sdAbs include high solubility, thermal stability, and low immunogenicity to humans [12]. A number of sdAbs have also been developed to treat a spectrum of human diseases, and some are currently in the late stages of clinical trials. For example, two antibodies, ozoralizumab (an anti-tumor necrosis factor (TNF)-alpha sdAb) and ALX-0061 (an interleukin (IL)-6R sdAb), are also in phase II trials [13].

The efficacy of CsA on animal models for RA is so far not satisfactory, even paradoxical [14]. A few other CypA inhibitors, such as the CsA derivative SDZ NIM811, capable of inhibiting neutrophil influx *in vivo* have been reported [5]. However, previous studies focused on the ability of CypA to regulate chemotaxis, and did not investigate other critical functions of CypA, such as the stimulation of MMP secretion that leads to cartilage destruction. Until now, there have been no reports of CypA-specific antibodies used for the treatment of RA. In this study, we characterized a new sdAb that was shown to inhibit important biological functions of CypA both *in vitro* and *in vivo*.

## Materials

### Animals and patient samples

The DBA/1 mice and the NOD/SCID mice were purchased from Shanghai Laboratory Animal Co. Ltd (Shanghai, China). Peripheral blood and synovial fluid were obtained from patients with active RA and the synovium tissues were obtained from RA patients undergoing joint replacement surgery or synovectomy. All of the RA patients met the 1987 revised diagnostic criteria of the American College of Rheumatology [15]. The normal human cartilage specimens were obtained from nonarthritis patients with femoral head fractures. Ethics approval was granted from the Ethics Committee of Fourth Military Medical University. All patients gave their informed consent to participate in this study. Likewise, all experiments involving animals were reviewed and approved by the Laboratory Animal Center of Fourth Military Medical University.

### Cells isolation and culture

The human monocytes were purified from peripheral blood of the RA patients using the Monocyte Negative Isolation kit (Invitrogen, Grand Island, NY, USA), and 1 × 10^6^ cells/ml were cultured in 2 ml RPMI 1640 with 10% fetal bovine serum (Gibco, Grand Island, NY, USA) with 15 ng/ml recombinant human macrophage colony-stimulating factor (R&D System, Emeryville, CA, USA) in six-well plates at 37°C. Macrophages were used after 7 days of culture. For synovial fluid of RA patients, monocytes/macrophages were isolated by Dynabeads® CD14 (Invitrogen) according to the manufacturer’s instructions.

The human monocytic THP-1 cells (American Type Culture Collection, Manassas, VA, USA) were cultured in RPMI 1640 medium supplemented with 10% fetal bovine serum at 37°C in 5% carbon dioxide. For the induction of cell differentiation, cells (5 × 10^5^ to 10^6^/ml) were stimulated with 100 nM phorbol 12-myristate 13-acetate (Sigma-Aldrich, St. Louis, MO, USA) for 48 hours.

### Library construction and single-domain antibody selection

Two adult male alpacas were immunized with endotoxin-free recombinant human CypA [9] as described previously [16]. Library construction and panning were performed as described previously [17]. Following three rounds of panning, individual clones producing target-binding sdAbs were identified by monoclonal phage enzyme-linked immunosorbent assay (ELISA). A horseradish peroxidase-labeled anti-M13 monoclonal antibody (GE Healthcare, Pittsburgh, PA, USA) diluted at 1:10,000 in phosphate-buffered saline (PBS) was used as a secondary antibody. The peroxidase enzyme activity was determined by adding TMB as a substrate, and the signal was read by optical density at 450 nm with a multiwell microplate reader (Bio Tek Instruments, Norcross, GA, USA).

Four positive clones were recloned into the modified expression vector pCANTAB5 His and were transformed into HB2151 cells. An overnight culture from a single colony was added to 2 × YT broth supplemented with 0.2% glucose and 100 μg/ml ampicillin. This culture was grown until the optical density at 600 nm reached 0.6 to 0.9. The expression of sdAb was induced with 1 mM isopropyl β-d-1-thiogalactopyranoside overnight at 30°C. Cells were then pelleted, resuspended and ultrasonicated. The sdAb protein fragments were purified by a combination of immobilized metal-ion affinity chromatography using His Trap HP metal affinity resin column (GE Healthcare) and size-exclusion chromatography with HiLoad 16/60 Superdex 75 prep grade column (GE Lifesciences, Pittsburgh, PA, USA). Protein was quantified with BCA Protein assay kit (Pierce, Rockford, IA, USA), with BSA used as a standard.

### Surface plasmon resonance measurements

The binding kinetics and affinity of sdAbA1 for purified CypA protein were obtained using the ProteON XPR36 protein interaction array system (Bio-Rad Laboratories, Hercules, CA, USA). A GLC chip was activated, on which recombinant CypA protein (10 μg/ml, diluted in 10 mM sodium acetate buffer, pH 4.5) was immobilized at 25°C in the vertical orientation. Following that, the remaining carboxyl groups were blocked with 1 M ethanolamine–HCl (pH 8.5). Finally, sdAbA1 (diluted in 10 mM phosphate buffer with 150 mM NaCl, pH 7.4) was injected in the horizontal orientation at five concentrations in twofold serial dilution down from 24 nM to 1.5 nM at a flow rate of 50 μl/minute. Antibody binding was evaluated by simultaneously flowing six sdAb concentrations that ranging from 0 to 24 nM over the CypA-coated chip for 180 seconds, and then monitoring dissociation for 720 seconds. The data were analyzed with ProteON Manager™ 3.1 software (Bio-Rad, Hercules, CA, USA), and binding constants were determined using a 1:1 Langmuir binding model.

### Peptidyl-prolyl cis–trans isomerase activity assay

The peptidyl-prolyl cis–trans isomerase (PPIase) activity was determined in a coupled assay with chymotrypsin using the tetrapeptide substrate Suc-Ala-Phe-Pro-Phe-*p*-nitroanilide (Sigma, St. Louis, MO, USA) [18]. All reagents were pre-equilibrated until the temperature reached 0°C. In a 1 ml cuvette, purified CypA was mixed with 10 μl chymotrypsin (20 μg/μl), and the volume was adjusted to 975 μl with assay buffer (50 mM HEPES, 100 mM NaCl, pH 8.0). The reaction was initiated by the addition of 25 μl tetrapeptide substrate at a final concentration of 37.5 μM. Changes in absorbance due to released *p*-nitroaniline were measured every 10 seconds for a maximum of 250 seconds in a spectrophotometer at 390 nm. Cleavage of the tetrapeptide substrate in the absence of CypA was used as a blank control, and the addition of CypA was used as a positive control for PPIase activity. CypA (PPIase inhibitor) was added at a concentration of 5.0 μM. The effect of sdAbA1 on the PPIase activity of CypA was performed with the pre-incubation of CypA with sdAbA1 for 2 hours before addition to the reaction system.

### Induction, treatment, and assessment of collagen-induced arthritis

The collagen-induced arthritis (CIA) model was constructed as described previously [19]. Male DBA/1 mice were immunized intradermally at the base of the tail with 200 μg chicken type II collagen (Chondrex, Redmond, WA, USA) emulsified in Freund’s complete adjuvant (BD Biosciences, San Jose, CA, USA) at the age of 8 to 12 weeks. On day 21 after primary immunization, mice were given a booster injection intradermally using the same concentration of type II collagen but with Freund’s incomplete adjuvant. Given that disease onset in this model varies widely for each mouse, and that not all mice develop arthritis, we in total used 100 mice to construct the CIA model. When immunized mice started to show clinical symptoms, 30 arthritic mice (an animal exhibited a clinical score in the range of 1 to 3 on day 27) were chosen to receive different treatments and were randomly assigned in cages. Ten mice were injected intraperitoneally with anti-CypA antibody sdAbA1 at a dose of 5 mg/kg once a day between days 27 and 37 after primary immunization. As a control, the other 20 mice were given intraperitoneal injections with PBS or isotype control (sdAbE2) at the same time points at which sdAbA1 was given. Clinical severity was scored on a scale of 0 to 4 as described previously [20]. The individual mouse arthritic score was obtained by summing the scores recorded for each limb. All clinical evaluations were performed by two investigators who were unaware of treatment group to which the mouse belonged.

### Assessment of radiographic joint damage in collagen-induced arthritis

X-ray radiographs were taken (In Vivo Imaging System FX PRO, Carestream, Riverside, CA, USA) of the hind paws of mice that had been given a general anesthetic. The degree of bone erosion in the ankle joint was scored as described previously [21] (0 = no swelling or bone damage, 1 = mild bone damage, 2 = moderate bone erosion, and 3 = severe bone erosion). The severity of arthritis in each mouse was determined independently and blindly by two observers.

### Construction of the SCID-HuRAg model

The synovium tissues were obtained from six patients with RA judged by American College of Rheumatology criteria. None of the patients included in the study had received treatment with corticosteroids or disease-modifying anti-rheumatic drugs. The SCID-HuRAg model was constructed as described previously [22]. In brief, 6-week-old to 8-week-old male NOD/SCID mice were used in this study. A 1 cm incision was made in the left flank. Normal human cartilage and rheumatoid synovial tissue were placed in the chamber in the muscle using fine forceps. The entire procedure was performed under sterile conditions.

### Single-domain antibody A1 treatment

Four weeks after implantation, 24 mice were randomly divided into four different treatment groups and were randomly assigned to cages. SdAbA1 (5 mg/kg, *n* = 6), infliximab (10 mg/kg, *n* = 6), sdAbE2 (5 mg/kg, *n* = 6) or PBS (*n* = 6) was administered three times a week within the implanted tissue using a micro-syringe. The injections were repeated over 4 weeks. The mice were anaesthetized and euthanized 7 days after the final injection for the removal of the implanted tissue.

### Histologic evaluation of SCID-HuRAg mice

The implanted tissues were removed from the SCID-HuRAg mice, paraffin embedded, and stained with hematoxylin and eosin for morphological evaluation. The number of inflammatory cells per unit was counted using Image-Pro Plus 6.0 (Media Cybernetics, Silver Spring, MD, USA). With this system, cells with similar characteristics (such as shape and size) can then be counted. Three measurements were made for each unit area, and the mean value was then calculated. The invasion of synovial tissue into the cartilage was quantified according to a semiquantitative score ranging from 0 to 4, based on the number of invading cell layers and the number of affected cartilage sites. Erosion was scored on a scale of 0 to 4 as described previously [23]. Cellular density was assessed on sections involving invasion and adjacent to the cartilage by counting the cells in three high-power fields at 400× magnification. Histological assessments were made under double-blind conditions. Three animal researchers recorded the data on separate case record forms without exchanging any information.

Immunohistochemical staining was performed using a streptavidin/peroxide kit (Zymed, San Francisco, CA, USA) according to the manufacturer’s instructions. The monoclonal antibody used was mouse anti-human MMP-9 antibodies (Santacruise, Dallas, TX, USA). Staining intensity was assessed on a semiquantitative five-point scale (0 = absent, 1 = weak, 2 = moderate, 3 = high, and 4 = very high). Histologic assessment was evaluated in a blinded manner by experienced pathologists.

### Serum cytokine detection by cytometric bead array

The blood of sacrificed mice was obtained by heart puncture 9 weeks after implantation and six human cytokines (TNFα, IL-1β, IL-6, IL-8, IL-10 and IL-12p70) in serum were analyzed simultaneously using a cytometric bead array (BD Biosciences) according to the manufacturer’s manual. Briefly, 50 μl of each sample or standard (0 to 5,000 pg/ml) were added into 50 μl mixtures (capture antibody-bead reagent and detector antibody-phycoerythrin reagent at a volume of 1:1) and incubated at room temperature for 3 hours away from light. After washing to remove the unbound detection reagent, the mixtures were then loaded onto flow cytometry and analyzed with cytometric bead array software.

### Gelatin zymogram

To study the influence of sdAbA1 on MMP secretion, gelatin zymogram was performed. Briefly, cells (1 × 10^6^ cell/well in 24-well plates) were starved for 24 hours, and then pretreated with sdAbA1, sdAbE2 or CsA for 2 hours before CypA stimulation. After treatment with CypA for 24 hours, the cell culture supernatants were collected. Each sample was resolved by SDS-PAGE under nonreducing conditions. The gels were then washed twice in 2.5% (v/v) Triton X-100 for 30 minutes at room temperature and incubated in reaction buffer for 16 hours at 37°C. The gels were subsequently stained with 0.5% Coomassie blue (R-250) and were destained to visualize the zymogen bands. The zymography gels were scanned and analyzed using US National Institutes of Health (Bethesda, MD, USA) Image 1.6 software.

### Cell chemotaxis inhibition assay

The mononuclear cells were obtained from heparinized venous blood by the Ficoll-Hypaque (Sigma) gradient centrifugation method. The chemotaxis assays were conducted as described previously [9]. Briefly, the mononuclear cells (1 × 10^6^ cells/ml) were added to the upper chamber of 48-well chemotaxis plates, while media containing CypA (100 ng/ml), *N*-formyl-Met-Leu-Phe (FMLP, used as a positive control) or medium alone were added to the lower compartments. The concentration of FMLP used was 10^–7^ M to induce optimal monocyte migration. For blocking experiments, sdAbA1 (20 μg/ml), sdAbE2 (20 μg/ml) or CsA (2 μM) was included in the lower wells. After incubation for 90 minutes, the number of cells appearing on the lower face of the filter was counted under microscope for each well, and each experimental condition was assayed in triplicate. A chemotactic index was calculated for each well by dividing the number of cells counted for that well by the number of cells in wells containing medium alone.

### Western blot analysis

THP-1 cells were plated in six-well plates and cultured in serum-free RPMI 1640 medium for 18 hours. Following this, the cells were pretreated with indicated concentrations of sdAbA1, sdAbE2 or CsA for 2 hours before CypA stimulation. For the mitogen-activated protein kinase inhibitor and nuclear factor NF-κB inhibitor experiment, the cells were pretreated with PD98059 (ERK1/2 inhibitor), 1-pyrrolidine carbodithioic acid, ammonium salt (PDTC, NF-κB inhibitor) or *N*-tosyl-l-phenyl-alanylchloromethyl ketone (TPCK, NF-κB inhibitor) for 2 hours before the addition of CypA. After stimulation with CypA for 24 hours, the cell culture supernatants were collected and tested in two ways. One part of the supernatant was used to assay the activity of MMP-9 by gelatin zymogram and by MMP-9 activity assay kit (Ray Bio Tech, Guangzhou, China) according to the manufacturers’ instructions. The other part of the supernatant was used to measure the level of IL-8 by a human CXCL8/IL-8 immunoassay kit (R&D System, Emeryville, CA, USA). The expression of IκBα and NF-κB P65 was observed 2 hours after CypA stimulation. The levels of ERK and phosphor-ERK1/2 were measured 30 minutes after CypA treatment. Briefly, the cells were harvested and the proteins were prepared with a commercial kit according to the manufacturer’s instructions (KeyGEN, Nanjing, China). In brief, cells were incubated in 10 volumes of hypotonic Buffer A on ice for 15 minutes and homogenized. Nuclei were recovered by centrifugation at 16,000 × *g* for 5 minutes, and the supernatant was collected as the cytosolic extracts. The nuclei were extracted using Buffer C for 40 minutes on ice. Insoluble material was removed by centrifugation at 16,000 × *g* for 10 minutes, and the supernatant was used as the nuclear extract. The extracts were then separated by SDS-PAGE and transferred to PVDF membrane (Millipore, Billerica, MA, USA ). Target bands were blotted with various primary antibodies (anti-phosphor-ERK1/2, anti-ERK1/2, anti-p65 and anti-histone) and horseradish peroxidase-conjugated secondary antibodies were used to develop the membrane.

### Statistical analysis

Data are presented as the mean ± standard error of the mean from three independent experiments unless otherwise indicated. All statistical analyses were performed using SPSS 15.0 statistical software (IBM SPSS, Chicago, IL, USA). Statistical analysis of the density of total MMP, inflammatory cell numbers, chemotactic index and cytokine concentrations was carried out using Student’s *t* test. In the CIA experiment, an independent-sample *t* test was used to compare the clinical severity between groups. Differences in cartilage invasion score, histologic data, and bone erosion score between the treatments groups were assessed by Kruskal–Wallis test followed by the Mann–Whitney U test.

## Results

### Generation and characterization of single-domain antibodies targeting cyclophilin A

**Figure 1:**
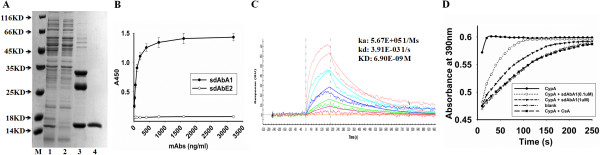
**Characterization and identification of a novel single-domain antibody targeting cyclophilin A. (A)** Expression and purification of anti-CypA single-domain antibody (sdAb) A1. The sdAbA1 antibody was purified from periplasmic lysates by Ni-NTA columns followed by gel filtration. The protein sample collected during purification was size fractionated by SDS-PAGE and stained with Coomassie blue. M, molecular weight markers, size indicated in kDa; lane 1, total proteins in periplasmic lysates as loaded onto the column; lane 2, flowthrough obtained from the Ni-NTA column; lanes 3 and 4, proteins eluted from the Ni-NTA column and Superdex 75 prep grade column respectively. **(B)** Binding of sdAbA1 to cyclophilin A (CypA) was detected by enzyme-linked immunosorbent assay. Purified CypA (1 μg/ml) was coated on 96-well plates and incubated with different concentrations of sdAbA1 or sdAbE2 (control sdAb). Binding of sdAbs was detected with horseradish peroxidase-conjugated anti-his antibody, visualized with TMB substrate and the plate was read at 450 nm. **(C)** Binding kinetics assay of sdAbA1 by surface plasmon resonance. **(D)** sdAbA1 inhibits the peptidyl-prolyl cis–trans isomerase (PPIase) activity of CypA using a chymotrypsin-coupled assay. CypA (0.1 μM) is shown as a positive control for PPIase activity and the absence of CypA and addition of CsA are used as blank control and negative control respectively. The presence of sdAbA1 inhibits the rate of cleavage of the tetrapeptide substrate in a dose-dependent manner compared with CypA alone. **(A)** to **(D)** Data are representative results from three independent experiments. ka, association rate constant; kd, dissociation rate constant; k_D_ the equilibrium dissociation constant .

A phage library of sdAbs was built from peripheral lymphocytes of the immunized animals and screened by the phage-display technique. After three rounds of panning, approximately 200 clones were picked out randomly to obtain the specific clones binding to CypA by phage ELISA. Four positive sdAbs with strong binding activities were obtained, expressed in *Escherichia coli* and purified. One of the isolated sdAbs, sdAbA1, appeared more capable of inhibiting cell migration and MMP secretion than the others and was further investigated in this study. The expression and purification of sdAbA1 in *E. coli* (HB2151) by immobilized metal affinity chromatography followed by gel filtration is shown in Figure 1A. The binding of sdAbA1 to recombinant CypA was further evaluated by ELISA, where sdAbE2, which had no detectable binding to CypA, was used as a negative control. As shown in Figure 1B, sdAbA1 displayed high levels of binding to recombinant CypA, while the control sdAbE2 exhibited little binding. The binding affinity of sdAbA1 for CypA was also determined by surface plasmon resonance, yielding a ka of 5.67 × 10^5^/M/second, a kd of 3.91 × 10^–3^/second and a calculated K_D_ of 6.9 × 10^–9^ M (Figure 1C).

Previous studies have demonstrated that the PPIase activity of CypA is crucial for its functions [24,25]. Here, we also detected the influence of sdAbA1 on the PPIase activity of CypA. The PPIase activity assay measures the efficiency of PPIase-catalyzed cis–trans isomerization of a commercially available tetrapeptide substrate that, following cis conversion to the trans isomer, is recognized and cleaved by chymotrypsin to result in yellow color formation. The addition of sdAbA1 significantly decreased the rate of tetrapeptide cleavage catalyzed by CypA in a concentration-dependent manner (Figure 1D).

### Effects of the anti-CypA single-domain antibody A1 on CIA

**Figure 2:**
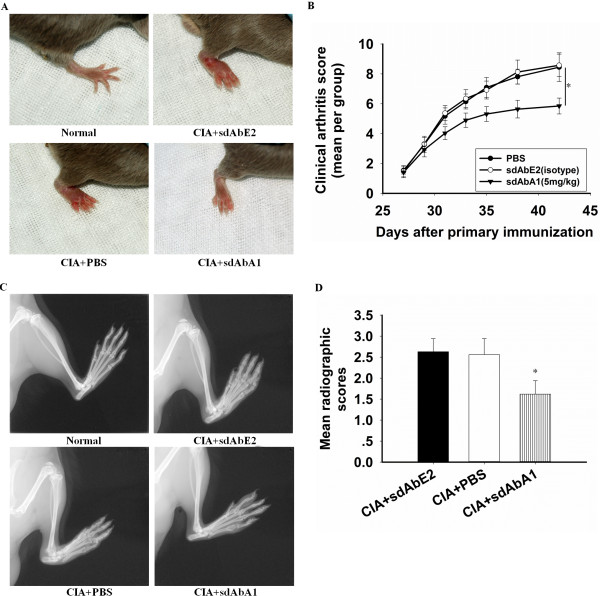
**Therapeutic administration of anti-CypA single-domain antibody A1 decreases arthritis severity in the collagen-induced arthritis model.** Mice with collagen-induced arthritis (CIA) were intraperitoneally injected with phosphate-buffered saline (PBS), isotype control antibody, or single-domain antibody (sdAb) A1 once a day from 27 to 37 days (*n* = 10 per group). Nonimmunized and nontreated animals were used as negative controls. **(A)** Representative photographs of the hind paw of CIA mice on day 42. **(B)** Clinical scoring was carried out as described in Materials and methods. sdAbA1 significantly reduced the clinical arthritis severity in CIA mice (*P <0.05 vs. control antibody group, for area under the curve). **(C)** Representative radiographs of the hind paw of CIA on day 42. **(D)** The degree of bone erosion on the radiographs of the hind paws of the mice was scored on a scale of 0 to 3 as described in Materials and methods. Values are the mean ± standard error of the mean of 10 mice per group. *P <0.05 versus control antibody group.

For CIA in mice, sdAbA1 was intraperitoneally injected at doses of 5 mg/kg without any toxic side effects. By analyzing each limb, a clinical score was obtained for the treated and control animals. Nonimmunized and nontreated animals did not develop any clinical signs of arthritis (Figure 2A, normal). Immunized animals treated with PBS or isotype control antibody served as positive controls and developed severe clinical signs of arthritis (Figure 2A, CIA + PBS and CIA + sdAbE2). Immunized animals treated with sdAbA1 showed a significant decrease of clinical signs of arthritis (*P* <0.05) (Figure 2B).

The severity of bone damage was examined radiologically in healthy mice and in mice with CIA under anesthesia. Joints in the hind paws were severely damaged in the PBS-treated or sdAbE2-treated control mice (Figure 2C,D, mean radiographic score 2.56 ± 0.38 in the PBS-treated group and 2.63 ± 0.31 in the isotype control-treated group). A significant inhibition of joint erosion could be observed in the mice injected with 5 mg/kg sdAbA1 (*P* < 0.05), and the mean radiographic score was 1.62 ± 0.32 (Figure 2D).

### Effects of single-domain antibody A1 on inflammatory cells in the implanted synovium and cartilage invasion

**Figure 3:**
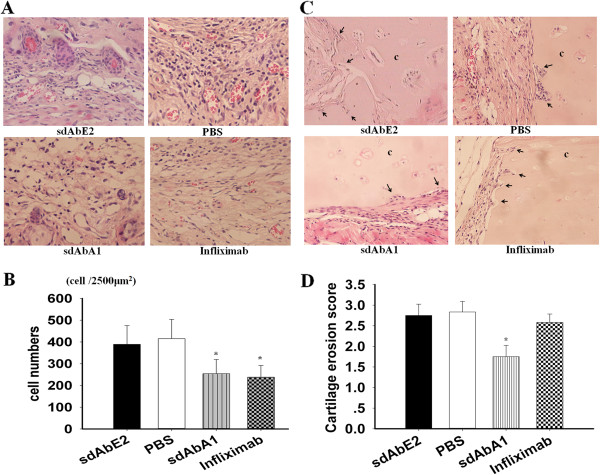
**Effects of single-domain antibody A1 on implanted synovium and cartilage erosion in SCID-HuRAg mice.** Rheumatoid arthritis (RA) synovium with normal human articular cartilage was co-implanted under the back muscle chamber of SCID-HuRAg mice. After 4 weeks of treatment, the co-implants were taken and stained with hematoxylin and eosin, and were evaluated histologically under a microscope. **(A)** Representative image of implanted synovium in SCID-HuRAg mice in each treatment group (*n* = 6 per group) (magnification × 400). **(B)** Statistical results of the numbers of inflammatory cells in each treatment group. **(C)** Representative images of human cartilage erosion in SCID-HuRAg mice of different groups (magnification × 200). **(D)** Cartilage erosion score after treatment in each group. Black arrows, invasive front of the synovial tissue. c, cartilage. *P <0.05 versus control antibody group. PBS, phosphate-buffered saline; sdAb, single-domain antibody.

We also evaluated the effects of sdAbA1 treatment on inflammation and cartilage destruction in the SCID-HuRAg mice. Infliximab, a widely used and highly effective treatment in RA, was applied as a positive control. The inflammatory cells were observed in the implanted tissues using hematoxylin and eosin staining (representative photographs in Figure 3A). The number of inflammatory cells in both the sdAbA1 treatment group (254 ± 66 cells) and the infliximab group (238 ± 54 cells) were significantly lower (*P* <0.05) than those of the control antibody group (389 ± 86 cells). No significant differences were observed between the control antibody and PBS-treated groups (415 ± 89) (shown in Figure 3B).

Histological results showed that the cartilage erosion was significantly less severe in the sdAbA1 group than in the infliximab group (erosion score 2.58 ± 0.20) or the control group (*P* <0.05). Deep invasion (invasion score ≥2.5) was observed in all six cartilage sections in the control group (Figure 3C). The mean erosion score in cartilage sections of the sdAbA1 group was 1.75 ± 0.27 (Figure 3D), compared with 2.75 ± 0.27 in the control antibody group (*P <*0.05; *n* = 6 per group). No significant differences were observed between the control antibody and PBS-treated groups (2.83 ± 0.26).

### Effects of single-domain antibody A1 on MMP-9 expression in the implanted synovium and inflammatory cytokines levels in the serum

**Figure 4:**
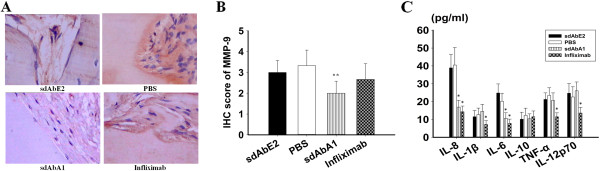
**Anti-CypA single-domain antibody A1 suppresses matrix metalloproteinase-9 and human inflammatory cytokine production in SCID-HuRAg mice. (A)** Representative images of matrix metalloproteinase (MMP)-9 staining of each treatment group under a microscope (*n* = 6 per group) (magnification × 400). **(B)** Statistical results for the expression of MMP-9 in each treatment group. **(C)** The blood of sacrificed mice was obtained and the levels of cytokines were detected by cytometric bead array (CBA). Results are expressed as the mean ± standard error of the mean with *n* = 6 per group. *P <0.05 compared with the control antibody group, **P <0.01 versus control antibody group. IHC, immunohistochemistry; IL, interleukin; PBS, phosphate-buffered saline; sdAb, single-domain antibody; TNF, tumor necrosis factor.

To clarify the mode and mechanism of action of sdAbA1, we assessed the effects of sdAbA1 on cytokine secretion and MMP expression. Immunohistochemical analysis demonstrated that treatment of mice with sdAbA1 significantly decreased the expression of MMP-9 compared with the PBS-treated group or the isotype antibody-treated group (Figure 4A,B) (*P <*0.01). Interestingly, there were no significant changes in MMP-9 production between the infliximab-treated group and control groups (*P >*0.05).

As shown in Figure 4C, the serum levels of the human inflammatory cytokines IL-6 and IL-8 were significantly lower in the sdAbA1 group than in the control antibody (*P <*0.05) and PBS-treated (*P <*0.05) groups. Notably, the levels of all tested cytokines (TNFα, IL-1β, IL-6, IL-8, and IL-12p70) were significantly lower in the infliximab group compared with the control antibody group. In this study, the amount of IL-10 was not shown any difference in different groups.

### Single-domain antibody A1 inhibits MMP-9 secretion and the chemotaxis induced by cyclophilin A

**Figure 5:**
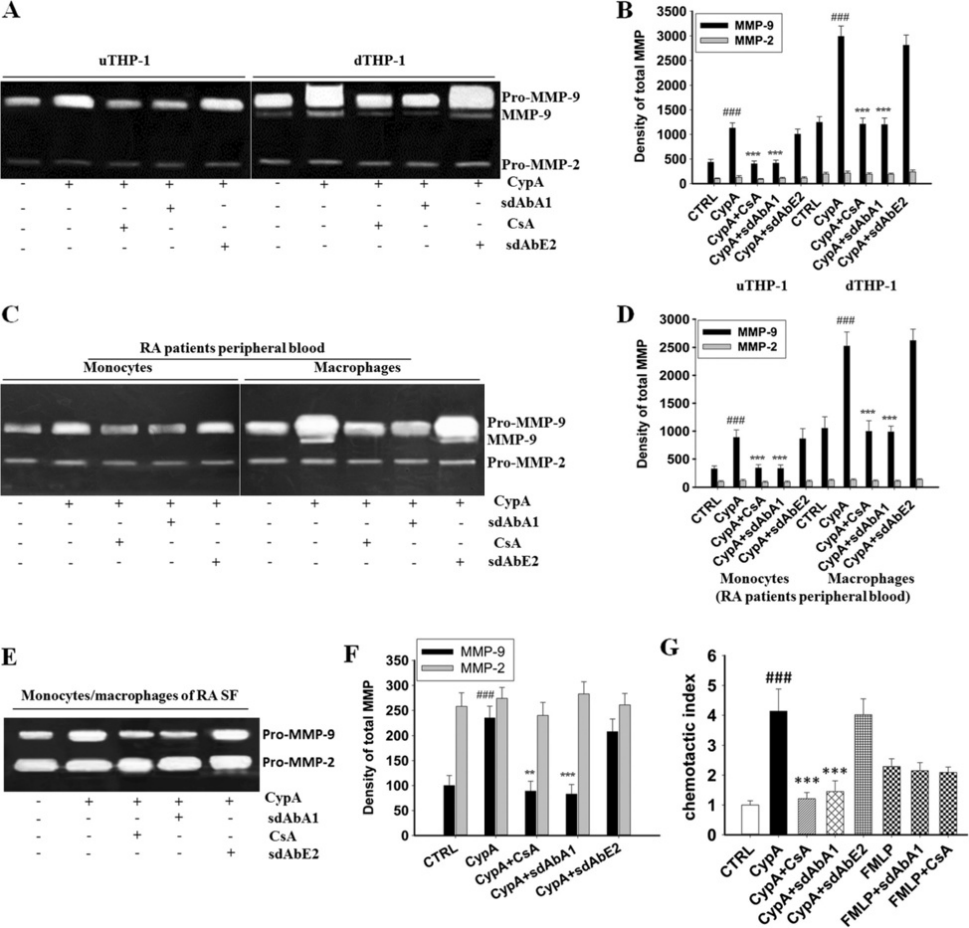
**Anti-CypA single-domain antibody A1 inhibits cell migration and matrix metalloproteinase production in human monocytes/macrophages.** The production of total matrix metalloproteinase (MMP)-9 and MMP-2 were tested by gelatin zymogram. **(A)**, **(C)** and **(E)** Representative photographs of gelatin zymogram using culture supernatants of monocytes/macrophages derived from THP-1, rheumatoid arthritis (RA) patients’ peripheral blood and RA synovial fluid (SF) respectively. **(B)**, **(D)** and **(F)** Statistical results of the density of total MMP-9 and MMP-2 produced by monocytes/macrophages derived from THP-1, RA patients’ peripheral blood and RA SF respectively. Data were results from three independent experiments. The protein expression of total MMP-9 (including Pro-MMP-9 and MMP-9) is significantly inhibited by single domain antibody (sdAb) A1 treatment. Each bar represents the mean ± standard error of the mean (SEM) of each group. **(G)** Cell migration mediated by cyclophilin A (CypA) is blocked by anti-CypA sdAbA1 antibody. Chemotaxis assays were set up using the peripheral mononuclear cells from RA patients incubated in the presence of a single dose of *N*-formyl-Met-Leu-Phe (FMLP, positive control) plus cyclosporine A (CsA) or sdAbA1, a single dose of CypA (100 ng/ml) plus sdAbA1, CsA or control antibody sdAbE2 (no binding to CypA). A chemotactic index was calculated for each group by dividing the number of migrated cells in test wells by the number of cells that migrated to medium alone. Bar graphs show mean ± SEM for each group, with *n* = 3 wells per group. ###*P* <0.001 versus control group; **P <0.01, ***P <0.001 versus CypA group. dTHP-1, differentiated THP-1; uTHP-1, undifferentiated THP-1.

To further study the mechanism of action of sdAbA1, we tested the effects of sdAbA1 on the MMP-9 secretion and cell chemotaxis induced by CypA using the monocytes/macrophages from RA patients. Firstly, to determine the impact of sdAbA1 on the ability of CypA to regulate MMP production, gelatin zymogram was performed. The representative photographs of gelatin zymogram using culture supernatants of monocytes/macrophages from THP-1, RA patients’ peripheral blood and RA synovial fluid are shown in Figure 5A,C,E, respectively. As shown in Figure 5D, the density of total MMP-9 in monocytes (888 ± 133) and macrophages (2524 ± 248) from RA patients’ peripheral blood with CypA stimulation was higher than that in the control group (329 ± 49 in monocytes, 1,052 ± 206 in macrophages; *P* <0.001), and decreased markedly when adding sdAbA1 (337 ± 58 in monocytes, 987 ± 103 in macrophages; *P <*0.001) or CsA (345 ± 54 in monocytes, 996 ± 192 in macrophages; *P <*0.001). Similarly, the density of total MMP-9 in monocytes/macrophages from RA synovial fluid with CypA stimulation (235 ± 23; *P <*0.001) was higher than that in the control group (100 ± 20), and reduced when adding sdAbA1 (83 ± 19; *P <*0.001) or CsA (89 ± 20; *P <*0.01) (Figure 5F). No changes were observed by the isotype antibody control sdAbE2 (*P* >0.05). Neither sdAbA1 nor CsA had any influence on pro-MMP-2 secretion (*P* >0.05). Since the THP-1 cells were selected for functional experiments, we also assayed the influence of sdAbA1 on MMP secretion in THP-1 cells under CypA stimulation. Similar to the results in the monocytes/macrophages from RA patients, the density of total MMP-9 in undifferentiated THP-1 (1,128 ± 106) and differentiated THP-1 (THP-1, 2,988 ± 210) cells with CypA stimulation was higher than that in the control group (434 ± 60 in undifferentiated THP-1, 1,245 ± 116 in differentiated THP-1; *P <*0.001), and was markedly reduced by adding sdAbA1 (415 ± 61 in undifferentiated THP-1, 1,199 ± 133 in differentiated THP-1; *P <*0.001) or CsA (403 ± 57 in undifferentiated THP-1, 1,208 ± 125 in differentiated THP-1; *P* <0.001) (Figure 5B). However, no significant changes were observed in the pro-MMP-2 secretion (*P >*0.05).

We then tested the effects of sdAbA1 on the cell chemotaxis induced by CypA using the RA patients’ peripheral mononuclear cells. The CypA chemotactic index for peripheral mononuclear cells (4.14 ± 0.74) was higher than that in the control group (1.00 ± 0.14, *P <*0.001). The chemotactic index decreased significantly when sdAbA1 or CsA was added (1.45 ± 0.36 and 1.21 ± 0.21, respectively; *P <*0.001) (Figure 5G). No significant differences in chemotactic index were observed among the groups treated with CypA alone versus those treated with CypA plus sdAbE2 (*P >*0.05). Importantly, neither sdAbA1 nor CsA had any effect on FMLP-induced migration of cells, demonstrating that inhibition was specific for CypA.

### Single-domain A1 counteracts the positive impacts of cyclophilin A on MMP-9 secretion and NF-κB activity via the ERK pathway

**Figure 6:**
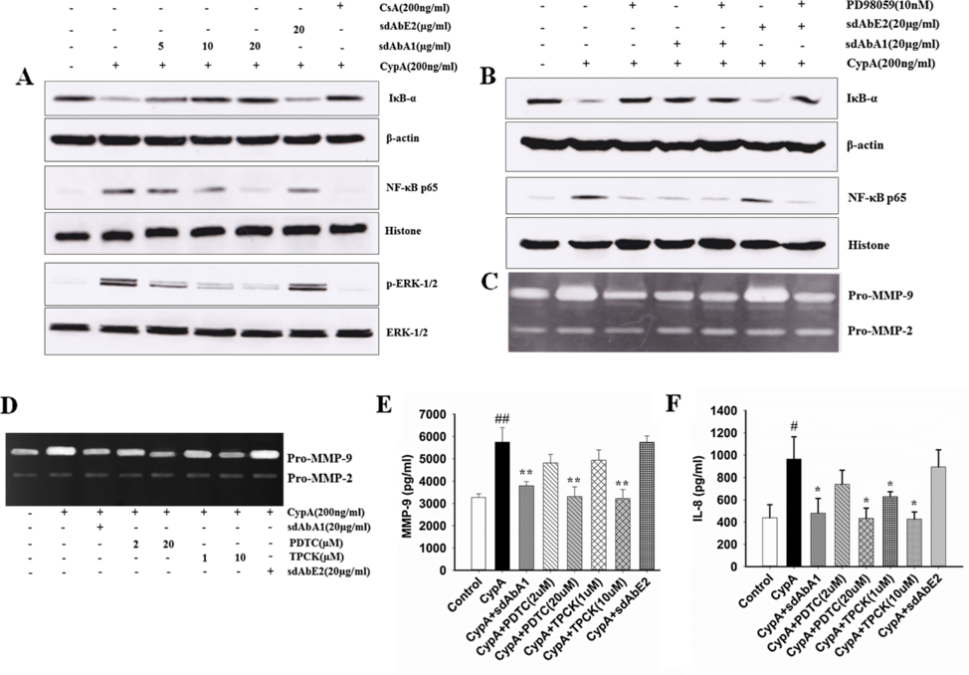
**Single-domain antibody A1 inhibits the production of MMP-9 and IL-8 stimulated by cyclophilin A through the ERK–NF-κB signaling pathway. (A)** THP-1 cells were pretreated with different concentrations of single-domain antibody (sdAb) A1 or cyclosporine A (CsA) for 2 hours before cyclophilin A (CypA) stimulation. The expression of cytoplasmic IκBα, nuclear factor NF-κB-p65 at 2 hours, the level of ERK1/2 protein and phosphorylation of ERK1/2 at 30 minutes were analyzed by western blot, while β-actin of cytoplasm protein and histone of nuclear protein were used as internal control respectively. **(B)** THP-1 cells were pretreated with PD98059 for 2 hours before adding CypA. The level of IκBα and NF-κB-p65 protein at 2 hours and matrix metalloproteinase (MMP)-9 activity at 24 hours after CypA stimulation were determined by western blot and **(C)** gelatin zymography separately. **(D)**, **(E)**, **(F)** THP-1 cells were pretreated with 1-pyrrolidine carbodithioic acid, ammonium salt (PDTC) and *N*-tosyl-l-phenyl-alanylchloromethyl ketone (TPCK) (two inhibitors of NF-κB) for 2 hours before adding CypA. After 24 hours, the culture supernatants were collected and the activity of MMP-9 determined using gelatin zymogram **(D)** and the Ray Bio Tech (Guangzhou, China) MMP-9 activity immunoassay kit **(E)** separately. The level of interleukin (IL)-8 in the culture supernatants was also tested by the R&D Systems (Emeryville, CA, USA) human CXCL8/IL-8 immunoassay kit **(F)** according to the manufacturer’s instructions. **(A)** to **(D)** Data are representative results from three independent experiments. **(E)**, **(F)** Bar graphs show the mean ± standard error of the mean for each group, with *n* = 3 wells per group. #*P* <0.05, ##*P* <0.01 versus control group; *P <0.05, **P <0.01 versus CypA group.

We tested whether the inhibitory effects of sdAbA1 on MMP-9 expression were dependent on NF-κB activation. As shown in Figure 6A, sdAbA1 treatment significantly decreased the degradation of cytoplasmic IκBα and translocation of NF-κB P65 into the nucleus stimulated by CypA in a dose-dependent manner. To further explore the upstream regulatory molecules leading to the inhibition of NF-κB, we analyzed the activities of the mitogen-activated protein kinases. Treatment with CypA combined with 5, 10, and 20 μg/ml sdAbA1 decreased the p-ERK1/2 level by 47.23 ± 3.45%, 61.64 ± 3.85%, and 74.25 ± 3.76%, respectively, compared with treatment with CypA alone. To demonstrate that sdAbA1 inhibits the activation of NF-κB through the ERK pathway, thus leading to decreases of MMP-9 production, PD98059 (an inhibitor of ERK) was used. No significant differences were observed in the degradation of cytoplasmic IκBα or the translocation of NF-κB P65 among the groups treated with sdAbA1, PD98059 or sdAbA1 associated with PD98059 (*P >*0.05) (Figure 6B). Similar results were observed in pro-MMP-9 secretion by gelatin zymography (Figure 6C). All of these results suggest that sdAbA1 was able to reverse the NF-κB activity and MMP-9 expression induced by CypA through the ERK pathway.

### Effects of NF-κB inhibitors on MMP-9 secretion and IL-8 expression

To further demonstrate that CypA-induced MMP-9 expression and IL-8 secretion requires NF-κB activation, THP-1 cells were pretreated with the TPCK and PDTC inhibitors of NF-κB before CypA stimulation. Both of these inhibitors significantly blocked the CypA-induced MMP-9 expression (Figure 6D) and IL-8 secretion (*P* <0.05; Figure 6F). As shown in Figure 6E, the levels of MMP-9 reduced dramatically on adding NF-κB inhibitors (*P* <0.01)*.* The amounts of IL-8 decreased significantly from 964.89 ± 199.26 pg/ml (CypA stimulation group) to 433.78 ± 91.00 pg/ml (PDTC, 20 μM) and 427.11 ± 63.65 pg/ml (TPCK, 10 μM), respectively (*P <*0.05). Moreover, we also observed in the sdAbA1 treatment group that the production of IL-8 decreased markedly (*P <*0.05 in Figure 6F) compared with that in the CypA treatment group, indicating that sdAbA1 was able to decrease IL-8 secretion induced by CypA through inhibiting the activation of NF-κB*.*

## Discussion

Although treatments for RA targeting TNFα or IL-1β have proved effective for many patients, there are still some problems to be solved – such as the nonuniversal adequacy and maintenance of response and risks of adverse effects like infection and malignancy. New targets in the treatment of RA therefore need to be investigated.

CypA, a widely distributed intracellular protein, was secreted by cells in response to inflammatory stimuli. Extracellular CypA is now commonly referred to as a cytokine involved in several different inflammatory diseases, including RA. CypA played an important role in the pathogenesis of human RA [6-9], as well as in CIA [26], thus providing an attractive target for therapeutic interventions. In the present study, we identified a novel anti-CypA antibody sdAbA1 and demonstrated its therapeutic potential for RA. Treatment with sdAbA1 ameliorated arthritis severity and joint destruction in two different models for RA. This was associated with reductions in inflammatory cell numbers, MMP expression, and proinflammatory cytokines that are well known for their critical role in inducing inflammation and bone erosion.

It is well known that each animal model cannot reflect the complexity of human disease. In order to evaluating the effects of the novel anti-CypA antibody sdAbA1 on RA, two different animal models of RA, the CIA model and the SCID-HuRAg mouse model, were adopted. Since the disease onset in CIA model varies widely for each mouse, 100 mice were used to construct the model. When mice showed clinical symptoms, 30 arthritic mice with a clinical arthritis score (1 to 3 on day 27) were chosen to receive different treatments. The initial disease severity of these arthritic mice is similar, and thus the results could more readily reflect the true efficacy of different treatments. A significant decrease in the clinical arthritis score as well as joint damage was observed in the sdAbA1 group. In the SCID-HuRAg model, our data showed remarkable reductions in the number of inflammatory cells and the degree of cartilage erosion in the sdAbA1-treated group compared with the control group. Moreover, sdAbA1 treatment prevented cartilage destruction more effectively than the drug infliximab.

It has been well established that proinflammatory cytokines and MMPs are involved in the pathogenesis of RA [2,3]. CypA may stimulate macrophages to degrade joint cartilage via MMP-9 expression and promote inflammation via proinflammatory cytokine production [7,8]. In this study, immunohistochemical staining revealed that the MMP-9 expression in the grafts was remarkably lower in the sdAbA1-treated group compared with the control group. The ability of sdAbA1 to inhibit MMP-9 production may explain why sdAbA1 exhibited stronger anti-erosion effects than the anti-TNFα monoclonal antibody. In addition, the serum levels of IL-6 and IL-8 in the SCID-HuRAg model were significantly reduced following sdAbA1 treatment. These findings indicate that sdAbA1 exerts anti-inflammatory and anti-joint damage effects on RA by inhibiting MMP-9 expression and secretion of IL-6 and IL-8.

Although it is largely unclear how CypA is involved in chemotaxis and MMP-9 regulation, one critical mechanism occurs via the interaction of CypA with one of its receptors, EMMPRIN (CD147) [27-29]. A study reported that fibroblast-like synoviocytes from CIA mice secreted CypA and enhanced CD147 expression in macrophages [30]. In RA patients, upregulated expression of CD147 was also found on circulating and synovial monocytes/macrophages [31], and high levels of CypA were also detected in the synovial fluid [6]. These reports suggest that an interaction in the synovial joints between extracellular CypA and CD147 expressed by macrophages may be a mechanism involved in the development of RA. Interestingly, we found that sdAb1 could block the CypA–CD147 interactions by carrying out competitive ELISA (data not shown). To further clarify the mechanism of the therapeutic effects of sdAbA1 on RA, CypA was added into monocytes/macrophages from RA patients *in vitro* to mimic an *in vivo* environment of rheumatic joints. Our results showed that cell migration and MMP-9 secretion of RA patients’ monocytes/macrophages were remarkably inhibited by sdAbA1. Moreover, western blot results demonstrated that sdAbA1 was able to reverse the NF-κB activity induced by CypA through the ERK pathway, thus leading to downregulation of MMP-9. In order to explore the relationship between NF-κB activation and MMP-9 production, the two NF-κB inhibitors TPCK and PDTC were used. This method showed that the levels of MMP-9 reduced dramatically by adding NF-κB inhibitors. Other studies also suggested that CD147 mediate the effects of extracellular CypA via inducing the activation of NF-κB [8,32], which was consistent with our results. These studies therefore suggested that sdAbA1 may block the interaction between CypA and CD147 and inhibit NF-κB activation through ERK1/2, thus leading to the downregulation of MMP-9.

Furthermore, IL-8 secretion stimulated by CypA was reduced by adding to either sdAbA1 or NF-κB inhibitors *in vitro*. However, other studies have reported that knockdown of CD147 does not result in change in CypA-mediated stimulation of IL-8, suggesting that CD147 is not the only cellular receptor of extracellular CypA [32]. The exact mechanisms behind the IL-8 inhibition by sdAbA1 treatment remain to be further elucidated. Interestingly, our results indicate that NF-κB may be involved.

## Conclusions

In summary, we identified a novel sdAbA1 that neutralizes CypA, which decreases both cartilage/bone erosion and synovial inflammation in two different animal models through inhibition of the ERK–NF-κB pathway. This characterization of the anti-CypA sdAbA1 could help to develop new strategies for the control of RA.

## Abbreviations

CIA: collagen-induced arthritis; CsA: cyclosporine A; CypA: cyclophilin A; ELISA: Enzyme-linked immunosorbent assay; FMLP: *N*-formyl-Met-Leu-Phe; IL: Interleukin; MMP: Metalloproteinase; NF-κB: Nuclear factor-κB; PBS: phosphate-buffered saline; PDTC: 1-pyrrolidine carbodithioic acid, ammonium salt; PPIase: Peptidyl-prolyl cis–trans isomerase; RA: rheumatoid arthritis; sdAb: Single-domain antibody; TNF: Tumor necrosis factor; TPCK: *N*-tosyl-l-phenyl-alanylchloromethyl ketone.

## Competing interests

The authors declare that they have no competing interests.

## Authors’ contributions

LW participated in the design of the study and drafted the manuscript. JFJ carried out the gel zymography assay and the chemotaxis assay, and performed the statistical analysis. CHW constructed the *in vivo* assays in the CIA model. XKM performed the *in vivo* assays in the SCID-HuRAg model. CGL assisted with the immunization of alpacas and antibody library construction. ZGF participated in the screening of the library. BW performed the expression, purification and identification of the antibody protein. XMY carried out the cytokine assay. YL participated in the study design and data interpretation. PZ participated in the design of the study and helped to draft the manuscript. ZNC conceived the study, and overviewed data analysis and interpretation. All authors read and approved the final manuscript.

## Authors’ information

LW, JFJ, CHW and XKM are co-first authors.
